# Determinants of low birth weight in Franceville, Southeast Gabon

**DOI:** 10.11604/pamj.2024.47.218.40737

**Published:** 2024-04-29

**Authors:** Yann Vital Sima Biyang, Cyrille Bisseye, Mahmoudou Saidou, André N´Tchoreret

**Affiliations:** 1 Laboratoire de Biologie Moléculaire et Cellulaire, Université des Sciences et Techniques de Masuku, Franceville, Gabon,; 2 Centre Hospitalier Universitaire Amissa Bongo, Franceville, Gabon; 3 Centre de Recherches Médicales de Lambaréné, Lambaréné, Gabon

**Keywords:** Low birth weight, risk factors, Franceville, Gabon

## Abstract

**Introduction:**

birth weight is a critical indicator of neonatal health and predicts future developmental outcomes. Despite its importance, there is a notable lack of research on the determinants of low birth weight (LBW) in southeast Gabon. This study aims to fill this gap by identifying factors contributing to LBW at the Centre Hospitalier Universitaire Amissa Bongo in Franceville.

**Methods:**

this retrospective analysis covered the period from February 2011 to May 2017, focusing on postpartum women and their infants. Data were analyzed using R software (version 4.3.2), employing both descriptive statistics and logistic regression. Statistical significance was determined at a p-value of less than 0.05.

**Results:**

among the 877 births analyzed, the prevalence of LBW was 8.4%. Bivariate analysis identified several factors associated with an increased risk of LBW, including, primigravida women (COR (95%CI) =0.59 (0.36-0.98), P = 0.036), primiparous women (COR (95%CI) =0.58 (0.36-0. 94), P = 0.024), women with a gestational age <37 weeks (COR (95%CI) =0.07 (0.04-0.11), P<0.001), women with ≤2 antenatal visits (COR (95%CI) =0.39 (0.18-0.93), P= 0.021), and women who underwent cesarean delivery (COR (95%CI) =0.46 (0.26-0.84), P = 0.008). However, multivariate analysis showed that only gestational age (AOR (95%CI) = 0.07 (0.04-0.11), P<0.001) and cesarean delivery (AOR (95%CI) = 0.48 (0.25-0.95), P = 0.03) were significantly associated with LBW.

**Conclusion:**

this study highlights the importance of gestational age and delivery method in the prevalence of LBW in southeast Gabon. These findings underscore the need for targeted interventions to address these risk factors, thereby improving neonatal health outcomes.

## Introduction

Low birth weight (LBW), a significant global public health issue, affects around 17% of live births. This problem is more pronounced in developing countries, where the incidence is approximately 19%, compared to 7% in developed nations [1,2]. LBW is defined as a birth weight of less than 2,500 grams, regardless of the gestational age at birth [3]. The primary causes of LBW include preterm delivery (before 37 completed weeks of gestation) and intrauterine growth restriction [4], both of which can have detrimental effects on the short, medium, and long-term health and development of the infant [5]. Consequently, LBW is a critical indicator of neonatal survival and future health outcomes [6]. Despite the prevalence of LBW being documented at 12-13% in previous studies within Gabon [7,8], there is a lack of specific data concerning the factors contributing to LBW in Franceville, a southeastern urban area of the country. Therefore, this study aims to assess the factors associated with LBW among newborns at the maternity ward of the *Centre Hospitalier Universitaire* Amissa Bongo in Franceville, located in the Haut-Ogooue province.

## Methods

**Study design and settings**: this retrospective study, conducted from February 2011 to May 2017, aimed to explore the correlation between maternal and newborn factors and low birth weight at the *Centre Hospitalier Universitaire* Amissa Bongo in Franceville. Franceville serves as the capital of the Haut-Ogooué province in the southeastern region of Gabon, which shares its border with the Republic of Congo.

**Participants**: the study population comprised postpartum women and their newborns, with data extracted from birth registers. Maternal variables analyzed included socio-demographic and economic information, gestational age, gravidity, parity, weeks of amenorrhea, number of antenatal visits, mode of delivery, hemoglobin levels (indicative of anemia), and malaria status. Neonatal variables assessed were the Apgar scores at 1- and 5-minutes post-birth and birth weight. Newborns with a birth weight below 2,500 g were classified as cases, measured to a precision of 10 grams using a mechanical scale. Controls were identified as newborns with a birth weight of 2,500 g or more. Inclusion criteria were limited to women with singleton pregnancies who had complete records on the variables of interest. Exclusions were applied to twin pregnancies and instances where data were missing.

**Statistical analysis**: the collected data from participants were recorded in a Microsoft Excel spreadsheet, ensuring accurate and organized data management. For the purpose of conducting a comprehensive statistical analysis, we utilized R software, version 4.3.2, known for its robustness in handling complex statistical computations and data visualization. To compare categorical variables, we employed two statistical tests based on the data distribution and sample size: the Chi-square test was used for variables with larger sample sizes, providing a reliable measure of association between categorical variables, while Fisher's exact test was utilized for smaller sample sizes or when the assumptions of the Chi-square test were not met, ensuring accuracy in the analysis of categorical data. The evaluation of the impact of socio-demographic characteristics of mothers on the incidence of low birth weight in newborns was conducted using a bivariate logistic regression model. This approach allowed us to examine the relationship between each independent variable and the outcome variable (low birth weight), controlling for one variable at a time. The results from this model are presented as odds ratios (ORs) with their respective 95% confidence intervals (CIs), offering a measure of the strength and direction of the association between variables. For a more comprehensive understanding of the factors contributing to low birth weight, a multivariate logistic regression model was employed. This model included variables that demonstrated significant associations in either the univariate analysis or the bivariate logistic regression model. The inclusion of these variables allowed for the assessment of their independent effects on low birth weight while controlling for other factors in the model. Statistical significance in this multivariate model was determined by a p-value of less than 0.05, adhering to the conventional threshold for statistical significance.

**Ethical consideration**: this study received approval from the Ethical Committee of the *Centre Hospitalier Universitaire* Amissa Bongo, ensuring that all research activities were conducted in accordance with ethical standards and principles. This approval underscores the commitment to respecting the rights, dignity, and welfare of the participants involved in the study.

## Results

**Sociodemographic characteristics of mothers and newborns**: the study examined a total of 877 postpartum women, with an average age of 26.2±6.8 years. The age group between 21 and 35 years was the most well-represented, accounting for 64% (558 out of 877) of the participants (Table 1). The majority of the women had no income, making up 68% of the sample. Women living with a partner constituted the largest group, comprising 46% of the participants. The study findings revealed that most women were multigravida (73%) and multiparous (66%). Nearly 95% of them had attended at least three prenatal visits (Table 1). Vaginal deliveries were the most common, accounting for 87% of all births, and 95% of the mothers had no medical history. However, the study estimated that 13% of deliveries were preterm. Additionally, 41% of women experienced anemia during pregnancy, while 15% had malaria infection (Table 1). Regarding the newborns, the study recorded a total of 877 births, with the majority being male (52%). The rate of low-birth-weight babies was 8.4%, with an average weight of 2148±321g. Furthermore, 4.3% of newborns had an Apgar score below 7. Among the low-birth-weight babies, 59.5% were preterm, while 40.5% were hypotrophic.

**Table 1:** maternal and newborn characteristics (N=877)

Variable		Number (N)	Percentage (%)	95%CI
Age Groups	≤20 yrs	223	25.0	22.6-28.5
	21-35 yrs	558	64.0	60.3-66.8
	≥36 yrs	96	11.0	9.0-13.2
Occupation	With income	278	32.0	28.6 - 34.9
	Without income	599	68.0	65.1 - 71.4
Marital status	Single	350	40.0	36.7-43.2
	Concubinage	401	46.0	42.4-49.1
	Married	126	14.0	12.1-16.9
Gravidity	primigravidae	240	27.0	24.5-30.5
	Multi-gravidae	637	73.0	69.5-75.5
Parity	Primiparous	298	34.0	30.9-37.2
	Multiparous	579	66.0	62.8 - 69.1
Gestational age	<37 weeks	116	13.0	11.1 - 15.7
	≥37 weeks	761	87.0	84.3-88.9
Prenatal visit	≤2	44	5.0	3.7-6.7
	≥3	833	95.0	93.3 - 96.3
Delivery type	Normal	764	87.0	84.7-89.2
	Cesarean	113	13.0	10.8-15.3
Medical history	No	829	95.0	92.8 - 95.9
	Yes	48	5.5	4.1 - 7.2
Anemia	No	359	41.0	37.7-44.3
	Yes	518	59.0	55.7-62.3
Malaria	No	749	85.0	82.9 - 87.6
	Yes	128	15.0	12.4 - 17.1
Newborns sex	Female	425	48.0	45.1-51.8
	Male	452	52.0	48.2 - 54.9
Newborns weight	LBW	74	8.4	6.7 - 10.5
	NBW	803	91.6	89.5-93.3
Apgar score	<7	38	4.3	3.1 - 6.0
	≥7	839	95.7	94 - 96.9

LBW= low birth weight; NBW= normal birth weight

**Risk factors associated with low birth weight**: univariate, bivariate, and multivariate analyses were conducted to identify significant associations between various variables and low birth weight. The study findings have been presented in Table 2 and Table 3. The results of the univariate analysis indicate that several factors were significantly associated with low birth weight. Maternal age of 20 years or less (P=0.035), being a primigravida (P=0.035), being a primipara (P=0.023), having a gestational age of less than 37 weeks amenorrhoea (P<0.001), having fewer than two antenatal visits (P=0.026), and undergoing a caesarean delivery (P=0.007) were all found to be significantly associated with low birth weight. However, no significant association was found between social status, marital status, medical history, anaemia, malaria, Apgar score, and low birth weight (Table 2). The bivariate analysis indicated that women who were primigravida (COR 95%CI = 0.599, (0.36-0.98), P = 0.036), primiparous (COR 95%CI = 0.58, (0.36-0.94), P = 0.024), and had a gestational age of less than 37 weeks amenorrhea (COR 95%CI = 0.07 (0.04-0.11), P < 0.001) were at risk of giving birth to low-birth-weight infants. In Table 2, the results demonstrated a significant association between low birth weight and gestational age (COR 95%CI = 0.07 (0.04-0.11), P < 0.001), having less than or equal to 2 antenatal visits (COR 95%CI = 0.39 (0.18-0.93), P = 0.021), and being born via caesarean section (COR 95%CI = 0.46, (0.26-0.84), P = 0.008). Furthermore, the multivariate analysis in Table 3 revealed that only gestational age (AOR 95%CI = 0.07 (0.04-0.12), P = 0.001) and cesarean delivery (AOR 95%CI = 0.48, (0.25-0.95), P = 0.03) remained significantly associated with low birth weight in newborns.

**Table 2:** risk factors associated with low birth weight in Franceville children (Univariable and bivariable model)

Variables		Univariable analysis			Bivariate model		
		LBW (n = 74)	NBW (n = 803)	p-value	COR	95%CI	p-value
Age Group	≤20	27 (36%)	196 (24%)	**0.035**	0.84	0.37, 1.77	0.666
	21-35	37(50%)	521(65%)		1.64	0.75, 3.30	0.188
	≥36	10(14%)	86(11%)		1	-	-
Occupation	With income	20(27%)	258(32%)	0.367	1	-	-
	Without income	54(73%)	545(68%)		0.78	0.45, 1.31	0.368
Marital status	Single	30(41%)	320(40%)	0.975	0.92	0.42, 1.88	0.826
	Concubinage	34(46%)	367(46%)		0.93	0.42, 1.88	0.848
	Married	10(14%)	116(14%)		1	-	-
Gravidity	primigravidae	28(38%)	212 (26%)	**0.035**	0.59	0.36, 0.98	**0.036**
	Multi-gravidae	46(62%)	591 (74%)		1	-	-
Parity	Primiparous	34(46%)	264(33%)	**0.023**	0.58	0.36, 0.94	**0.024**
	Multiparous	40(54%)	539(67%)		1	-	-
Gestational age	<37	44 (59%)	72(9.0%)	**< 0.001**	0.07	0.04,0.11	**< 0.001**
	≥37	30 (41%)	731(91%)		1	-	-
Prenatal visit	≤2	8 (11%)	36 (4.5%)	**0.026**	0.39	0.18, 0.93	**0.021**
	≥3	66(89%)	767 (96%)		1	-	-
Delivery type	Normal	57(77%)	707(88%)	**0.007**	1	-	-
	Cesarean	17(23%)	96 (12%)		0.46	0.26, 0.84	**0.008**
Medical history	No	72(97%)	757(94%)	0.421	1	-	-
	Yes	2(2.7%)	46(5.7%)		2.19	0.66,13.6	0.285
Anemia	No	25(34%)	334(42%)	0.191	1	-	-
	Yes	49(66%)	469(58%)		0.72	0.43, 1.17	0.193
Malaria	No	62(84%)	687(86%)	0.68	1	-	-
	Yes	12(16%)	116 (14%)		0.87	0.47, 1.75	0.68
Baby Sex	Female	30(41%)	395(49%)	0.154	1	-	-
	Male	44(59%)	408(51%)		0.7	0.43, 1.14	0.156
Apgar score	<7	6(8.1%)	32(4.0%)	0.125	0.47	0.20, 1.28	0.103
	≥7	68 (92%)	771 (96%)		1	-	-

CI= confidence interval; Crude odds ratios (OR) and 95% confidence intervals; significant association (P<0.05)

**Table 3:** risk factors associated with low birth weight in Franceville children (Multivariate model)

Variables		Multivariate model		
		AOR	95%CI	p-value
Age Group	≤20	1.36	0.45, 4.02	0.577
	21-35	1.85	0.77, 4.17	0.152
	≥36	1	-	-
Gravidity	primigravidae	0.76	0.25, 2.12	0.613
	Multi-gravidae	1	-	-
Parity	Primiparous	0.86	0.33, 2.58	0.779
	Multiparous	1	-	-
Gestational age	<37	0.07	0.04, 0.12	**< 0.001**
	≥37	1	-	-
Prenatal visit	≤2	0.73	0.30, 1.97	0.508
	≥3	1	-	-
Delivery type	Normal	1	-	-
	Cesarean	0.48	0.25, 0.95	**0.03**

AOR= adjusted odds ratio, CI= confidence interval; adjusted odds ratios (OR) and 95% confidence intervals (variables found to be associated through a univariate analysis or Bivariate model were entered into the multivariate model); significant association (P<0.05).

## Discussion

The primary goal of this study was to pinpoint the risk factors linked to low birth weight (LBW) among newborns in the maternity ward of the *Centre Hospitalier Universitaire* Amissa Bongo in Franceville. This urban center, nestled in Gabon´s southeastern expanse, was scrutinized through the lens of 877 recorded births, unveiling an LBW prevalence of 8.4%. This statistic is consistent with figures reported in Syria [9] and developed nations [10], yet it marks a distinct decrease from earlier studies in Gabon [7,8] and neighboring Central African regions like Cameroon (20.79% [5]) and the Republic of Congo (13.27% [3]). The documented 8.4% prevalence of LBW infants in Franceville indicates a notable deviation from the higher rates observed in both regional and rural Gabonese contexts. This discrepancy might be attributed to the superior quality of prenatal and maternal-infant health services offered in the area. The study recorded an average weight of 2148±321 g for underweight children, closely mirroring the findings from an Ethiopian study [11], which reported an average LBW of 2133.5±332.88 g. Crucially, the research identified gestational age below 37 weeks and cesarean deliveries as significant predictors of LBW, aligning with global research that emphasizes the heightened risk of LBW associated with preterm births [12,13]. These insights were further validated by a Brazilian study [14], which underscored the tenfold increased risk of LBW in preterm infants compared to their full-term counterparts. In a contrast from existing literature, this investigation found no significant association between LBW and factors such as maternal age, primigravidae, primiparity, the frequency of antenatal visits, delivery mode, socioeconomic or marital status, medical history, anemia, malaria, or Apgar scores. This divergence challenges the conclusions of prior studies [4,15-17], suggesting a nuanced interplay of determinants influencing LBW, potentially unique to the studied locale.

**Limitations of the study**: it is crucial to acknowledge the constraints of this study, primarily stemming from the limited number of participants. Moreover, it is imperative to recognize that the results solely apply to a specific geographical area within Gabon and should not be generalized to a wider population.

## Conclusion

Franceville´s relatively low incidence of LBW a stark contrast to prior Gabonese and regional research highlights the effectiveness of its health care system in managing prenatal care and safeguarding maternal-infant health. While gestational age and cesarean delivery emerged as significant LBW factors, the absence of correlations with other explored variables underscores the complexity of LBW aetiology. Further investigations, ideally with more extensive cohorts, are essential for corroborating these findings and enriching the global understanding of LBW risk factors.

### What is known about this topic

- *Low birth weight is a prevalent issue in sub-Saharan Africa, significantly impacting neonatal health and development;*
- *Research within Gabon has historically highlighted a high prevalence of low birth weight in children, particularly in rural regions;*
- *Before this investigation, comprehensive data on low birth weight in Franceville, an urban area in southeastern Gabon, was sparse*.

### What this study adds

- *This research uncovers a significant reduction in the prevalence of low birth weight in Franceville, documenting a rate of 8.4%; this finding marks a notable improvement compared to previous reports from rural areas within Gabon and similar contexts in sub-Saharan Africa;*
- *The study identifies only two primary risk factors associated with low birth weight: cesarean delivery and a gestational age of less than 37 weeks; these findings emphasize the importance of focusing on these aspects within prenatal care programs to mitigate the risk of low birth weight;*
- *Contrary to prior studies, this research did not establish any significant links between low birth weight and other commonly considered maternal or infant health risk factors; this discrepancy suggests that the dynamics influencing low birth weight in Franceville may differ from those in other regions, warranting a targeted approach to healthcare interventions in this area*.
